# Systematic *In Vitro* Evaluation of a Library of Approved and Pharmacologically Active Compounds for the Identification of Novel Candidate Drugs for KMT2A-Rearranged Leukemia

**DOI:** 10.3389/fonc.2021.779859

**Published:** 2022-01-20

**Authors:** Mawar Karsa, Emma Ronca, Angelika Bongers, Anna Mariana, Ernest Moles, Timothy W. Failes, Greg M. Arndt, Laurence C. Cheung, Rishi S. Kotecha, Maria Kavallaris, Michelle Haber, Murray D. Norris, Michelle J. Henderson, Lin Xiao, Klaartje Somers

**Affiliations:** ^1^ Children’s Cancer Institute, Lowy Cancer Research Centre, University of New South Wales (UNSW) Sydney, Sydney, NSW, Australia; ^2^ School of Women’s and Children’s Health, University of New South Wales (UNSW) Sydney, Sydney, NSW, Australia; ^3^ Australian Cancer Research Foundation (ACRF) Drug Discovery Centre for Childhood Cancer, Children’s Cancer Institute, Lowy Cancer Research Centre, University of New South Wales (UNSW) Sydney, Sydney, NSW, Australia; ^4^ Australian Research Council (ARC) Centre of Excellence in Convergent Bio-Nano Science and Technology and Australian Centre for Nanomedicine, University of New South Wales (UNSW) Sydney, Sydney, NSW, Australia; ^5^ Leukaemia Translational Research Laboratory, Telethon Kids Cancer Centre, Telethon Kids Institute, Perth, WA, Australia; ^6^ Curtin Medical School, Curtin University, Perth, WA, Australia; ^7^ Department of Clinical Haematology, Oncology, Blood and Marrow Transplantation, Perth Children’s Hospital, Perth, WA, Australia; ^8^ Division of Paediatrics, School of Medicine, University of Western Australia, Perth, WA, Australia; ^9^ University of New South Wales (UNSW) Centre for Childhood Cancer Research, University of New South Wales (UNSW) Sydney, Sydney, NSW, Australia

**Keywords:** *KMT2A*/*MLL*-rearranged leukemia, infant leukemia, repurposing, high-throughput screen, NR5A1, apoptosis

## Abstract

Patients whose leukemias harbor a rearrangement of the *Mixed Lineage Leukemia* (*MLL*/*KMT2A*) gene have a poor prognosis, especially when the disease strikes in infants. The poor clinical outcome linked to this aggressive disease and the detrimental treatment side-effects, particularly in children, warrant the urgent development of more effective and cancer-selective therapeutics. The aim of this study was to identify novel candidate compounds that selectively target *KMT2A*-rearranged (KMT2A-r) leukemia cells. A library containing 3707 approved drugs and pharmacologically active compounds was screened for differential activity against KMT2A-r leukemia cell lines versus KMT2A-wild type (KMT2A-wt) leukemia cell lines, solid tumor cells and non-malignant cells by cell-based viability assays. The screen yielded SID7969543, an inhibitor of transcription factor Nuclear Receptor Subfamily 5 Group A Member 1 (NR5A1), that limited the viability of 7 out of 11 KMT2A-r leukemia cell lines including 5 out of 7 lines derived from infants, without affecting KMT2A-wt leukemia cells, solid cancer lines, non-malignant cell lines, or peripheral blood mononuclear cells from healthy controls. The compound also significantly inhibited growth of leukemia cell lines with a *CALM-AF10* translocation, which defines a highly aggressive leukemia subtype that shares common underlying leukemogenic mechanisms with KMT2A-r leukemia. SID7969543 decreased KMT2A-r leukemia cell viability by inducing caspase-dependent apoptosis within hours of treatment and demonstrated synergy with established chemotherapeutics used in the treatment of high-risk leukemia. Thus, SID7969543 represents a novel candidate agent with selective activity against *CALM-AF10* translocated and KMT2A-r leukemias that warrants further investigation.

## Introduction

The conventional drug discovery path involves several drug development stages, from compound discovery, through to extensive preclinical drug optimization and characterization, which includes Absorption, Distribution, Metabolism, Elimination and Toxicity (ADMET) and efficacy studies in animal models, culminating in rigorous testing in humans through clinical trials. Due to this time-consuming process, it has typically taken more than a decade for new agents to advance from bench to bedside, with the vast majority of compounds failing to reach the final stages of clinical trial testing. As a result, an alternative and potentially more effective discovery approach based on drug repurposing has recently gained traction. Drug repurposing entails the use of drugs for indications other than those for which they were originally intended and therefore bypasses many development and optimization steps, shortening the timeline and decreasing the overall costs for drug approval.

Here we aimed to apply a drug repurposing approach to identify novel candidate drugs for targeting leukemias harboring a rearrangement of the *MLL* gene, recently renamed *KMT2A*, present in approximately ten percent of patients with acute leukemia (1–3). In general, the presence of this translocation is associated with an aggressive disease course, chemoresistance, an increased risk of relapse and poor prognosis, especially in the setting of acute lymphoblastic leukemia (ALL) in children below the age of one (1, 4, 5). Infant KMT2A-rearranged (KMT2A-r) ALL is one of the most challenging pediatric cancers with less than 40% of patients surviving 5 years past diagnosis (5, 6). Toxicity from current treatment, involving the use of intensified chemotherapeutic drug combinations and hematopoietic stem cell transplant, can be lethal in a significant percentage of patients and for those who survive, long-term detrimental health effects are common (5). More potent and safer treatment options for KMT2A-r leukemia, particularly for infant KMT2A-r ALL, are urgently needed.

To identify novel candidate compounds that selectively target KMT2A-r leukemia cells, we screened a library comprising of US Food and Drug Agency (FDA)-approved drugs and pharmacologically active compounds with known targets against a KMT2A-r leukemia cell line with counter screening against a KMT2A-wildtype (KMT2A-wt) leukemia cell line. We identified SID7969543, an inhibitor of transcription factor Nuclear Receptor Subfamily 5 Group A Member 1 (NR5A1), as a novel selective candidate inhibitor against a subset of KMT2A-r and *CALM-AF10* translocated leukemia cells, including cells derived from infants with KMT2A-r leukemia.

## Materials and Methods

### Chemicals and Reagents

SID7969543 was purchased from Tocris Bioscience (Bristol, United Kingdom). 2-Cl-ATP was procured from Santa-Cruz Biotechnology (Texas, USA). 2-CADO and cladribine were purchased from Sigma Aldrich (New South Wales, Australia). For the generation of lipid-based nanoparticles used to transfect leukemia cells with siRNA, cholesterol and the lipids 1,2-distearoyl-sn-glycero-3-phosphocholine (DSPC), 1,2-dimyristoyl-rac-glycero-3-methoxypolyethylene glycol-2000 (DMG-PEG), and 1,2-dioleoyl-sn-glycero-3-phosphoethanolamine-N-[lissamine rhodamine B sulfonyl] (DOPE-Rho) were obtained through Merck & Co., Inc. D-Lin-MC3-DMA was purchased from Assay Matrix Pty Ltd. siGENOME Human *NR5A1* siRNA (M-003429-00-0010) targeting NR5A1 and On-TARGETplus control pool non-targeting scrambled siRNA (D-001810-10-20) were purchased from Millennium Science Pty Ltd.

### Cell Lines and Cell Culture

All cell lines used in this study were mycoplasma-free and have been authenticated using STR profiling. Cells were cultured as previously described (7, 8). Characteristics of the leukemia cell lines used in this study are described in **Supplementary Table 1**. Peripheral blood mononuclear cells from healthy donors were purchased from Australian Red Cross.

### High-throughput Phenotypic Screening

The following chemical libraries were used in the screening as previously described (9): Prestwick Chemical Library (Prestwick Chemical PC SAS, France), LOPAC^®^1280 library (Sigma-Aldrich, Australia), Tocriscreen Plus library (Tocris Bioscience, UK) and Selleck Inhibitor Library (Selleck Chemicals, USA), together combining 3707 compounds. PER-485 (KMT2A-r) and CCRF-CEM (CEM, KMT2A-wt) leukemia cells were seeded into 384-well culture plates. Test compounds were added to the assay plates for a final concentration of 5 μM (in DMSO stock). Plates were incubated for 72 hours at 37°C, 5% CO_2_. Resazurin was added and plates were incubated for 7 hours. The difference in relative fluorescence units at time zero and 7 hours of resazurin incubation was calculated for each well. The percentage cell viability for each test compound was calculated relative to the cells treated with vehicle (100% viability).

### Cytotoxicity, Synergy, and *In Vitro* Cell-Based Assays

The cytotoxicity of drugs was assessed through resazurin-based assays and the inhibitory concentration resulting in 50% reduction of cell survival relative to control (IC_50_) was calculated as previously described (7). In synergy studies, cells were exposed to a dilution series of compounds, as single agents or in combination, in resazurin-based assays (72 hours) as described (8–10). The occurrence of synergy was determined with the Bliss Independence model as previously described (8–10). Bliss Prediction curves indicated the predicted percentage viability of the cells when exposed to the combination of compounds if both compounds work additively together. Synergy was visualised as the presence of a lower cell viability upon combination of two compounds compared to the viability predicted based on the presence of an additive effect of the compounds (i.e., the viability curve of the combination runs below the Bliss Prediction curve). The percentage of apoptotic cells was assessed by annexin V and 7-Aminoactinomycin D (7-AAD) (BD Biosciences, Australia) flow cytometry as previously described (7).

### Western Blot

Western blotting experiments were performed as previously described (7). Antibodies used are listed in **Supplementary Table 2**. Actin was used as a loading control.

### RNA Isolation and qRT-PCR

Total RNA was isolated using a Qiagen RNeasy kit and cDNA synthesized using the iScript cDNA Synthesis Kit (BioRad). Quantitative PCR was run and analyzed on Applied Biosystem’s QuantStudio5 Real-Time PCR Detection System, using Taqman gene expression assays for genes NR5A1 (Hs00610436_m1), GUSB (Hs99999908_m1) and HPRT (Hs99999909_m1) Relative expressions were determined as previously described (7).

### Mouse Liver Microsomal Stability Assays

Compound half-life was estimated by mouse liver microsomal stability assays as described (10).

### Synthesis of Lipid-Based Nanoparticles Encapsulating siRNA

Lipid-based nanoparticles encapsulating siRNA targeting NR5A1 and scrambled siRNA (LNP-siRNA) were prepared using a NanoAssemblr™ Spark^®^ instrument (Precision Nanosystems) as previously described (11). Prior to addition to cell cultures, LNP-siRNA suspensions were further diluted in PBS (pH 7.4) or culture media to lessen traces of ethanol to non-toxic levels (<0.5% vol/vol). Dynamic light scattering using a Malvern Zetasizer Ultra (Malvern Ltd) was used to determine the LNP-siRNA size (~67 nm) and polydispersity index (<0.2). siRNA encapsulation (%) in LNPs was determined by Quant-iT™ RiboGreen™ RNA Assay (ThermoFisher Scientific) in the presence and absence of Triton X-100 (0.5% vol/vol).

### LNP-siRNA-Mediated NR5A1 Silencing

Cells were seeded at 0.5 x 10^6^ cells/mL in 12 wells culture plates and incubated at 37°C, 5% CO2, with LNPs encapsulating anti-NR5A1 siRNA or scrambled siRNA at 250 nM siRNA concentration for up to 72 hours.

## Results

### Cell-Based Screening of FDA-Approved Drugs and Pharmacologically Active Compounds to Identify Selective Inhibitors of KMT2A-r Leukemia

**Figure 1:**
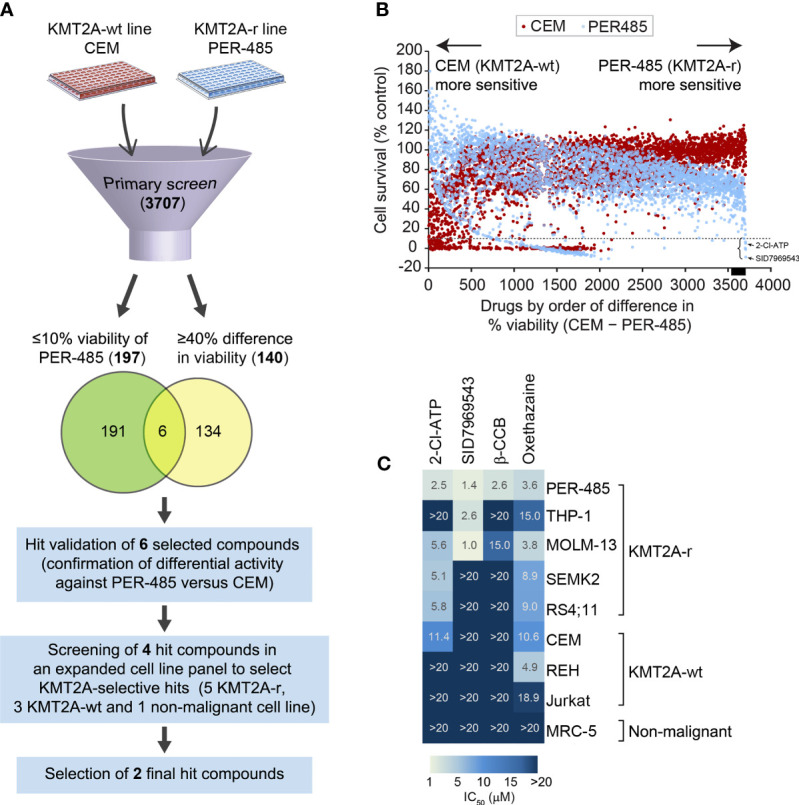
Cell-based screening for identification of selective inhibitors of KMT2A-r leukemia. **(A)** Schematic of the drug screen. **(B)** Percentage viability of KMT2A-wt CEM and infant KMT2A-r PER-485 cell lines represented by a dot plot after primary screening of the 3707-compound library. Each dot represents the viability of the specified cell line (CEM, red; PER-485, blue) after treatment with an agent (5 μM) from the compound library. Compounds are arranged on the x-axis according to the difference in growth inhibition of PER-485 cells compared to CEM cells (%viability of CEM – %viability of PER-485). Compounds showing higher inhibitory effects in PER-485 than in CEM are displayed towards the right of the x-axis, whereas compounds that CEM are more sensitive to compared with PER-485 are towards the left. The dotted line indicates 10% cell viability. The black bar on the x-axis indicates the position of the 140 drugs that have ≥40% difference in viability (%viability of CEM – %viability of PER-485 ≥40%). The left curly bracket indicates the position of the six hit candidates selected for further evaluation in expanded cell line panels. The black arrows indicate the achieved PER-485 %viability values corresponding to the two final hit compounds selected from the screen. **(C)** Heatmap of concentration with 50% cell growth reduction (IC_50_) achieved in different cell lines after treatment with 5 μM of each compound for 72 hours.

To identify novel compounds targeting KMT2A-r leukemia, a phenotypic cell-based viability screen on a library containing 3707 compounds performed previously was re-analyzed (9). The library, consisting of the Prestwick (n=1200), Tocris (n=1119), LOPAC (n=1280) and Selleck (n=108) libraries, comprised a mixture of drugs approved by the FDA and other agencies, such as the European Medicines Agency, and biologically active compounds that cover a wide range of targets including kinases, neurotransmitter receptors and G-protein-coupled receptors. The library was screened against a KMT2A-r leukemia cell line, PER-485, harboring the most common *KMT2A* translocation found in infants and pediatric patients [t(4;11)], and a KMT2A-wt leukemia cell line CCRF-CEM (9, 12, 13). Both lines were isolated from children with relapsed leukemia, providing excellent *in vitro* models for high-risk disease. The viability of cells treated with a fixed concentration (5 µM) of the library compounds was assessed in resazurin-based cytotoxicity assays after a 72-hour incubation to allow selection of drugs acting rapidly on cell viability as previously described (9). To discover potent compounds with selective cytotoxicity towards the KMT2A-r leukemia cells, we defined two stringent criteria to select hit compounds: 1) decreasing cell viability of PER-485 cells to 10% or less, and 2) producing a greater growth inhibition of PER-485 KMT2A-r leukemia cells compared to CEM KMT2A-wt leukemia cells (%viability of CEM – %viability of PER-485 ≥ 40%). One hundred ninety-seven compounds decreased the viability of the PER-485 cells to 10% or less after a 72-hour incubation. One hundred and forty agents showed a viability difference of ≥ 40% in CEM versus PER-485 cells (**Figures 1A, B**). A total of six compounds fell into both categories and were subsequently retested in a secondary validation screen in triplicate (**Figure 1A**). Four of these agents were confirmed to preferentially target KMT2A-r leukemia cells, including the adenosine analog 2-chloroadenosine triphosphate (2-Cl-ATP), SID7969543, an inhibitor of NR5A1, β-carboline-3-carboxylate (β-CCB), an endogenous proconvulsant and anxiogenic benzodiazepine receptor ligand, and oxethazaine, a Na^+^ channel blocker (**Figure 1C**).

### Screening Against a Panel of Leukemia Cell Lines Uncovers Two Candidate Compounds that Selectively Target KMT2A-r Leukemia Cells

To investigate the selectivity of the four short-listed hit compounds towards KMT2A-r leukemia, cytotoxicity assays were performed in a leukemia cell line panel (n=8) that included the PER-485 and CEM cells used in the original screen, as well as four additional KMT2A-r cell lines (both ALL and acute myeloid leukemia (AML)) with either t(4;11) (n=2) or t(9;11) (n=2) *KMT2A* translocations, two KMT2A-wt leukemia cell lines and a non-malignant cell line MRC-5 (**Figure 1C** and **Supplementary Table 1**). While β-CCB displayed strong cytotoxic activity with a concentration of 2.6 µM needed to achieve 50% inhibition of cell survival (IC_50_) in PER-485 cells, confirming our results from the original screen, it was less potent against the other KMT2A-r leukemia cell lines tested (**Figure 1C**). Oxethazaine demonstrated cytotoxicity against several KMT2A-wt and KMT2A-r leukemia cell lines with limited selectivity towards KMT2A-r leukemia cells. However, the other two compounds, 2-Cl-ATP and SID7969543 demonstrated selective cytotoxic activity against a subset of KMT2A-r leukemia cells without inhibiting the KMT2A-wt leukemia cells or the non-malignant cell line, MRC-5 (**Figure 1C**). 2-Cl-ATP and SID7969543 were therefore selected for further evaluation.

### KMT2A-r Leukemia Cells Are Sensitive to Adenosine Analogs

**Figure 2:**
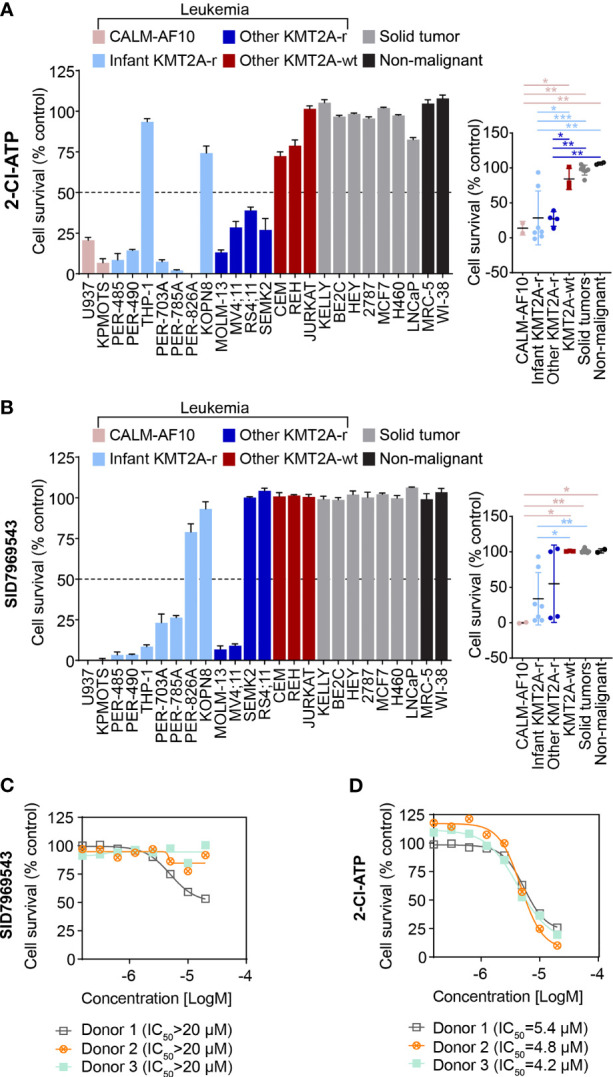
The adenosine analog 2-Cl-ATP and the NR5A1 inhibitor SID7969543 display selective cytotoxicity against a subset of KMT2A-r leukemia cell lines. The percentage viability of a 25-cell line panel after treatment with 10 µM 2-Cl-ATP **(A)** or SID7969543 **(B)** in a 72-hour resazurin-based cytotoxicity assay is represented in a bar chart (left). The panel on the right depicts the comparison of viabilities of cell lines (mean ± SE) grouped based on subtype: *CALM-AF10* translocated, infant KMT2A-r, other KMT2A-r, KMT2A-wt, solid tumor and non-malignant cell lines. Details of cell lines and IC_50_ are found in **Supplementary Table 1**. Dose response curves for peripheral blood mononuclear cells from three healthy donors when treated with SID7969543 **(C)** and 2-Cl-ATP **(D)** in a 72-hour resazurin-based viability assay. Statistical significance was determined by ANOVA with Dunn’s correction for multiple comparisons. Asterisks represent significance levels of P-values. *, *p*<0.05; **, *p*<0.01. ***, *p*<0.001.

To further characterize the cytotoxic profile of the adenosine analog 2-Cl-ATP, the compound was evaluated in an expanded leukemia cell line panel comprising six additional KMT2A-r leukemia cell lines, including five derived from infants with KMT2A-r ALL and two *CALM-AF10* translocated leukemias, which are KMT2A-wt but represent an aggressive leukemia subtype that shares underlying molecular etiological pathways with KMT2A-r leukemias, such as their dependency on DOT1L histone-lysine methyltransferase and an upregulation of *HOXA* cluster genes (14–16). In addition, to determine the specificity of the compound towards leukemia cells, we widened our cell line panel to incorporate non-leukemia cell lines, including seven solid tumor cell lines (neuroblastoma, breast cancer and lung carcinoma) and another non-malignant cell line, totaling 25 cell lines (KMT2A-r n=11, KMT2A-wt n=5, solid tumors n=7, non-malignant n=2) (**Supplementary Table 1**). 2-Cl-ATP showed inhibitory activity, with IC_50_ values below 10 µM, towards 9 out of 11 (82%) KMT2A-r leukemia cells with IC_50_ values ranging from 2.5 to 5.9 µM (**Figure 2A** and **Supplementary Table 1**). The compound also exerted significant inhibitory effects against both *CALM-AF10* translocated leukemia cell lines when compared to other KMT2A-wt leukemia cell lines, the solid cancer cell lines and non-malignant cells (**Figure 2A** and **Supplementary Table 1**). 2-Cl-ATP inhibited KMT2A-r leukemia cells by inducing apoptosis (**Supplementary Figure 1A**).

2-Cl-ATP is a phosphorylated derivative of the adenosine analog 2-chloroadenosine (2-CADO). Corroborating our observations, previous studies have reported sensitivity of KMT2A-r ALL cells to nucleoside analogs (17, 18). To further confirm that 2-Cl-ATP exerts a similar KMT2A-r selective action as other nucleoside analogs such as cladribine and that KMT2A-r leukemia cells are indeed susceptible to nucleoside analogs, we evaluated whether 2-Cl-ATP and 2-CADO had a similar cytotoxicity profile as cladribine in resazurin-based viability assays with a panel of KMT2A-r and KMT2A-wt leukemia cell lines (**Supplementary Figure 1B**). Indeed, a highly similar cell line selectivity was observed for all three drugs, with 2-Cl-ATP-resistant cell lines THP-1 and REH also being less responsive to 2-CADO and cladribine than the 2-Cl-ATP-sensitive KMT2A-r cells confirming our observed sensitivity of KMT2A-r leukemia cells to adenosine agonists.

### SID7969543 Rapidly Kills KMT2A-r Leukemia Cells by Inducing Apoptosis

The selectivity of SID7969543 towards KMT2A-r leukemia cells was also determined in the expanded cell line panel. Similar to 2-Cl-ATP, all cell lines that were sensitive towards SID7969543 (IC_50_ < 10 µM) were leukemias with either a *KMT2A* or *CALM-AF10* translocation (**Figure 2B** and **Supplementary Figure 2**). SID7969543 decreased the viability of a subset (7/11) of KMT2A-r cells as well as both *CALM-AF10* leukemia cells, with IC_50_ values ranging from 1 µM to 5 µM, while not affecting the other KMT2A-wt leukemia cells, solid tumors or non-malignant cells (**Figure 2B** and **Supplementary Figure 2**; **Supplementary Table 1**). Five of the seven sensitive KMT2A-r leukemia cell lines were derived from infants. No significant association was found between the occurrence of a certain *KMT2A* translocation and sensitivity to SID7969543. Both of the t(9;11), four out of six t(4;11) and one t(1;11) translocated KMT2A-r leukemia cell were sensitive to SID7969543, whereas the remaining two t(11;19) KMT2A-r leukemia cells did not display sensitivity (**Supplementary Table 1**). No significant associations were observed between sensitivity to SID7969543 and the presence of a lymphoid (ALL) or myeloid (AML) leukemia phenotype (**Supplementary Figure 3**). Remarkably, SID7969543 did not significantly impact the viability of peripheral blood mononuclear cells (PBMC) isolated from healthy donors at doses at which it significantly inhibited KMT2A-r leukemia cells (**Figure 2C**). This is in contrast to the inhibitory effects of 2-Cl-ATP against these PBMCs with IC_50_ values closer to those achieved against KMT2A-r leukemia cells (**Figure 2D**).

**Figure 3:**
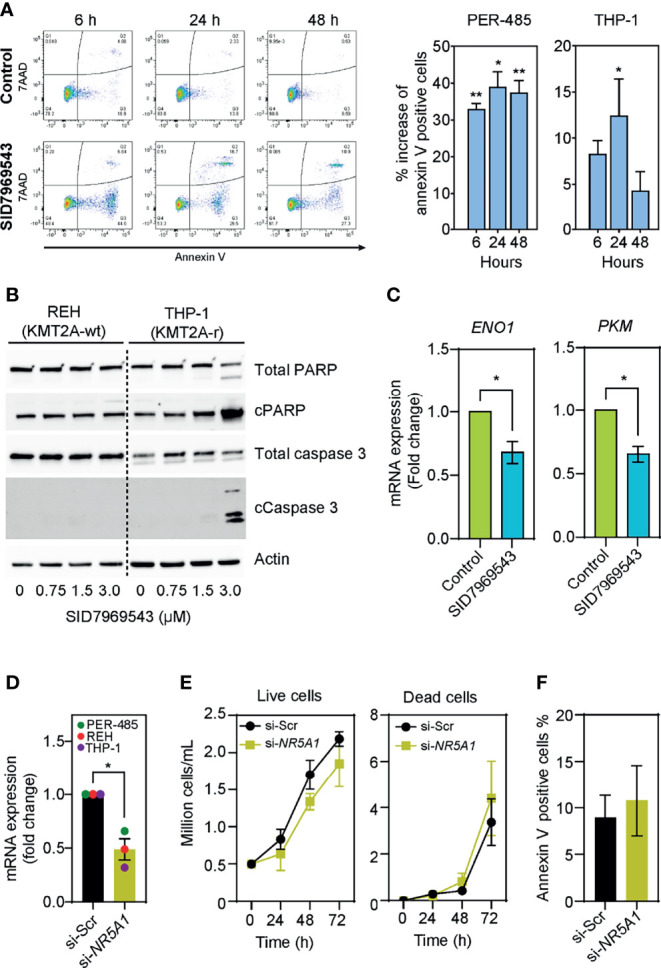
SID7969543 induces caspase 3-mediated apoptosis in KMT2A-r leukemia cells. **(A)** Representative flow cytometry plots of PER-485 cells (left) treated with the compound for up to 48 hours and stained for annexin V/7AAD. Quantifications (right) of mean percentage increases of annexin V positive PER-485 and THP-1 cells relative to vehicle-treated cells after treatment with their respective IC_50_ doses of SID7969543 (PER-485: 1.4 µM; THP-1: 2.6 µM). Statistical significance was determined by one sample t-test. **(B)** Western blots showing induction of apoptotic markers in KMT2A-r THP-1 cells but not in KMT2A-wt REH cells after treatment with SID7969543. **(C)** mRNA expression level of *ENO1* and *PKM* after treatment of KMT2A-r PER-485 cells with SID7969543 relative to vehicle-treated cells. Cells were treated with 1.4 µM (IC_50_ of for PER-485) of SID7969543 for three hours. **(D)** mRNA expression level of *NR5A1* after a 48-hour incubation of three KMT2A-r leukemia cell lines (PER-485, REH, and THP-1) with LNP-siRNA targeting NR5A1 (si-*NR5A1*) relative to cells treated with LNP encapsulating scrambled siRNA (si-Scr). **(E)** Cell growth of KMT2A-r PER-485 cells treated with si-Scr LNPs (control) or si-*NR5A1* up to 72 hours as determined by trypan blue exclusion cell count. **(F)** Percentage of annexin V positive PER-485 cells after a 48-hour treatment with si-Scr or si-*NR5A1* LNPs. Graphs depict mean ± SE of three independent experiments. Asterisks represent significance levels of P-values. *, *p*<0.05; **, *p*<0.01.

To identify how SID7969543 limited the viability of KMT2A-r leukemia cells, the percentage of cells expressing surface annexin V was quantified to evaluate induction of apoptosis. SID7969543 significantly increased the percentage of annexin V-positive KMT2A-r cells as early as 6 hours post-exposure (**Figure 3A**). This was associated with increased cleavage of PARP and caspase-3 in sensitive KMT2A-r, but not in KMT2A-wt leukemia cells (**Figure 3B** and **Supplementary Figure 4**), indicating that the compound rapidly kills sensitive KMT2A-r leukemia cells through caspase-dependent apoptosis.

To elucidate whether SID7969543 exerted its selective KMT2A-r leukemia cell killing effect through targeting and inhibiting its reported target, the transcription factor NR5A1, we firstly investigated whether the expression of *NR5A1* was associated with the occurrence of a *KMT2A* rearrangement. *NR5A1* expression was not significantly different between KMT2A-r and KMT2A-wt infant ALL patient samples (**Supplementary Figure 5A**) nor between KMT2A-r and KMT2A-wt leukemia cell lines (**Supplementary Figure 5B**), and the sensitivity of leukemia cell lines to SID7969543 did not correlate with expression levels of *NR5A1* (**Supplementary Figure 5C**). We next assessed whether the drug impacted downstream NR5A1 signaling in treated KMT2A-r leukemia cells. Treatment with SID7969543 significantly decreased the expression of reported NR5A1 target genes involved in metabolism, enolase-1 (*ENO1*) and pyruvate kinase (*PKM*) (19) in sensitive KMT2A-r cell lines before the induction of apoptosis, while the expression of genes not regulated by NR5A1 (e.g. the *KMT2A* target genes *HOXA9, HOXA10*, *MEIS1*, and *MYB*) remained unchanged, demonstrating that SID7969543 hit its reported target (**Figure 3C** and **Supplementary Figures 6, 7**). However, siRNA-mediated silencing of *NR5A1* expression did not selectively impact the viability of KMT2A-r leukemia cells and thus did not mimic the action of SID7969543 on these cells (**Figures 3D–F** and **Supplementary Figures 8, 9**). In addition, another structurally dissimilar inhibitor of NR5A1, the inverse agonist AC-45594, did not display selective cytotoxicity towards KMT2A-r leukemia cells (**Supplementary Figure 10**) (20). Taken together it is therefore unlikely that the sole inhibition of NR5A1 is causing the observed effects of SID7969543 on KMT2A-r leukemia cell survival.

### SID7969543 Synergizes With Conventional Chemotherapeutics for Childhood Leukemia

**Figure 4:**
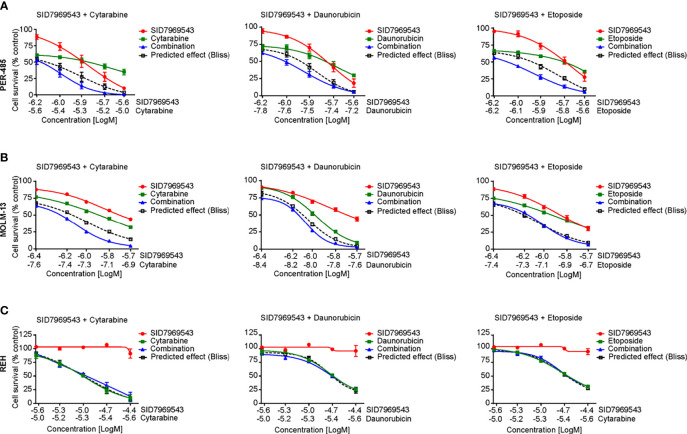
SID7969543 synergizes with conventional chemotherapeutics. Dose response curves for combination treatments of SID7969543 and standard-of-care chemotherapy drugs cytarabine, daunorubicin and etoposide in PER-485 **(A),** MOLM-13 **(B)** and REH **(C)** cells as determined by 72-hour resazurin-based viability assays. Graphs depict mean ± SE of three independent experiments. Drug synergy was calculated by applying the Bliss additivity model. Dotted lines indicate predicted viability if compounds are additive and combination curves below the dotted Bliss line indicate the occurrence of synergy between the tested drugs.

We next determined whether SID7969543 synergized with conventional chemotherapeutics used in the treatment of high-risk leukemia, namely, cytarabine, daunorubicin, etoposide, mitoxantrone, and topotecan (**Figure 4** and **Supplementary Figure 11**). Two KMT2A-r leukemia cell lines (PER-485, MOLM13) were treated with SID7969543, a conventional chemotherapeutic agent or their combination and potential synergistic effects were determined using the Bliss additivity model. For both cell lines, the viability curves of cells treated with the combination of SID7969543 and cytarabine or daunorubicin tracked below the predicted viability curves by Bliss (dotted lines), demonstrating synergy (**Figure 4**). For etoposide, synergy with SID7969543 was observed in the PER-485 cells, while the combination was additive in MOLM13 cells (**Figures 4A, B**). The combination of SID7969543 with mitoxantrone or topotecan had additive effects in both cell lines (**Supplementary Figures 11A, B**). No synergy between SID7969543 and cytarabine, daunorubicin or etoposide was found in KMT2A-wt REH cells (**Figure 4C**).

Taken together, these data demonstrate that SID7969543 rapidly kills a subset of KMT2A-r leukemia cells, particularly those arising in infants, *via* the induction of apoptosis. In addition, the compound potentiates several standard-of-care chemotherapies used for treatment of high-risk leukemia. However, microsomal stability assays showed that SID7969543 has a short half-life and thus limited *in vivo* stability (**Supplementary Figure 12**), precluding further testing in animal models in its current form.

## Discussion

KMT2A-r leukemias, which are particularly resistant to standard-of-care chemotherapy regimens, continue to be one of the most aggressive and difficult to treat leukemia types, especially in infants. Several novel agents such as DOT1L and FLT3 inhibitors that demonstrated selective targeting of KMT2A-r leukemia cells in preclinical models, have unfortunately failed to live up to their expectations in clinical trials (21, 22). While results of clinical trials testing the safety and efficacy of epigenetic regulators such as specific inhibitors of menin and histone deacetylases for application in KMT2A-r leukemia are awaited, the search for other potent drugs and novel targetable vulnerabilities remains ongoing (2, 23).

By performing a systematic *in vitro* evaluation of a library of approved drugs and pharmacologically active compounds, we have identified novel candidate compounds that selectively kill KMT2A-r leukemia cells *in vitro* within a short time frame, in contrast to slower acting epigenetic modifiers. The identification of the adenosine analog, 2-Cl-ATP as a selective inhibitor of KMT2A-r leukemia cell lines, is in line with previous studies highlighting the sensitivity of KMT2A-r leukemia to nucleoside analogs (17, 18) and supports the validity of our screening approach to identify KMT2A-r selective candidate drugs. Moreover, we demonstrate that this selective sensitivity to nucleoside analogs can be extended to *CALM-AF10* translocated leukemias.

SID7969543 preferentially inhibited the growth of a subset of KMT2A-r leukemia cells, which included various subtypes with different *KMT2A* translocations. No associations between the presence of certain *KMT2A* translocations or a lymphoid/myeloid phenotype of the leukemia cell lines and sensitivity to SID7969543 were observed in this study. Interestingly, we found that the majority of sensitive cell lines were derived from infants diagnosed at less than one year of age. Given the remarkable heterogeneity of KMT2A-r leukemia, these results are in line with infant KMT2A-r leukemia representing a unique disease subtype (24). While KMT2A-r leukemias are unified by an aggressive clinical disease course characterized by treatment resistance, other factors such as the specific *KMT2A* translocation, particular gene breakpoints, and age of the patient actually define different disease subtypes driven by diverse leukemogenic pathways (25–27). This also suggests the need to apply distinct therapeutic approaches or precision medicine for different subtypes of KMT2A-r leukemia. To achieve this, a comprehensive understanding of the complex heterogeneity of pathways involved in KMT2A-r leukemogenesis and leukemia progression is warranted to allow identification of targetable vulnerabilities for each patient. Our finding that SID7969543 also targets *CALM-AF10* translocated leukemia cells, supports previous reports showing that *KMT2A* and *CALM-AF10* translocations rely on common underlying leukemogenic pathways (14–16) and further emphasizes that both leukemia subtypes share common targetable vulnerabilities. This is in line with observations in our earlier studies that focused on large-scale chemical library screening and yielded novel candidate drug molecules with selectivity for KMT2A-r leukemia that were similarly cytotoxic against *CALM-AF10* translocated leukemias (7, 10). To be able to identify markers of responsiveness to SID7969543, such as the presence of certain molecular abnormalities (e.g., specific *KMT2A* translocations), a future larger scale screening in an expanded panel of KMT2A-r leukemia cell lines is warranted.

SID7969543 is an isoquinolinone derivative first identified in an ultra-high-throughput screen of approximately 65,000 compounds from the National Institute of Health’s Molecular Libraries Small Molecule Repository in a cell-based transactivation assay to search for potent and selective inhibitors of NR5A1 (28). NR5A1 is an orphan nuclear receptor and critical regulator for development of the adrenal cortex and gonads (29, 30). More recently, NR5A1 overexpression has been reported in adult and childhood adrenocortical carcinoma and has been shown to increase adrenocortical carcinoma cell proliferation, adrenocortical hyperplasia and tumor formation, suggesting that NR5A1 is involved in the regulation of cellular proliferation and maintenance (31–35). While the role of NR5A1 in regulation of the expression of genes involved in steroid hormone synthesis and cellular cholesterol homeostasis pathways is well defined, little is known about how this nuclear factor contributes to the development of cancer (36–38). In adrenocortical carcinoma, NR5A1 controls cellular proliferation and maintenance likely through pathways independent of steroidogenic gene regulation. Crosstalk between NR5A1 and transforming growth factor (TGF) β signaling, Wnt/β-catenin signaling as well as cell proliferation and macromolecule synthesis pathways including glycolysis, have been proposed to contribute to cancer progression (36, 38).

Only one other study has linked NR5A1 to hematological cancers; a recent large NGS short hairpin RNA knockdown screen identified *NR5A1* as a crucial gene for leukemic cell survival in one out of six primary AML samples (39), but the significance of this finding remains unclear. While our discovery of SID7969543 as a selective inhibitor of KMT2A-r leukemia highlights NR5A1 as a candidate target in KMT2A-r leukemia, the absence of a phenotypic effect of *NR5A1* silencing by siRNA and the lack of a similar KMT2A-r selective cytotoxicity of another structurally dissimilar inverse NR5A1 agonist suggests that it is unlikely that the inhibition of NR5A1 solely explains the selective killing of KMT2A-r leukemia cells by SID7969543. Of note, transient and incomplete silencing of NR5A1 by siRNA might not mimic the antagonistic effect of SID7969543 on NR5A1 and the alternative NR5A1 inhibitor, AC-45594, might have other targets that impact its cytotoxic selectivity. Further studies are needed to identify the mode of action of SID7969543 in KMT2A-r leukemia cells.

Structure-activity relationship (SAR) analysis with SID7969543 derivatives will be needed to identify the targeted pathway in KMT2A-r leukemia cells and develop clinically viable inhibitors. SAR analysis will pinpoint the functional groups essential for the KMT2A-r cell killing effect of the drug, guiding the development of analogs suitable for tagging and affinity chromatography to allow the identification of the molecular targets of SID7969543 and its derivatives in KMT2A-r leukemia cells. Silencing of candidate targets followed by downstream phenotypic analysis will subsequently provide insight into the identity of the true molecular target or targeted pathway for this class of compounds that mediates the observed KMT2A-r cell killing selectivity. These analyses will also direct the development of pharmacologically viable SID7969543 derivatives that have better drug-like properties and are suitable for evaluation in animal models of KMT2A-r leukemia.

While SID7969543 cannot be moved directly into the clinic, the strong *in vitro* findings of our study demonstrating the rapid killing of a subset of KMT2A-r leukemia cells, particularly those arising in infants, support further study of SID7969543 to identify its molecular target, develop drug-like derivatives and use as a tool compound *in vitro*.

Overall, our results show that SID7969543 represents a novel candidate agent with selective activity against *KMT2A* and *CALM-AF10* translocated leukemia, warranting further investigation.

## Data Availability Statement

The raw data supporting the conclusions of this article will be made available by the authors, without undue reservation.

## Author Contributions

MKar conducted the majority of the experiments with technical support from AB and ER. MKar, LX, and KS analyzed the data. AM, TF, and GA executed the drug screening at the Drug Discovery Centre, Children’s Cancer Institute Australia. EM generated lipid nanoparticles for silencing experiments. LC and RK provided guidance and access to cell lines used in the study. MKav, MH, and MN provided guidance and support with study design. MKar, MJH, LX, and KS designed the experiments. MJH and KS conceived the project. MJH and KS supervised the project. KS, MKar, and LX wrote the manuscript. All authors contributed to the article and approved the submitted version.

## Funding

This research was supported by a grant from Cancer Council NSW (Program Grant PG-1601) and philanthropic funds from the Tenix Foundation. For this work, MKar was supported by a PhD topup scholarship by CRC Cancer Therapeutics. KS received funding from the Kids Cancer Alliance. LX was supported by early-career researcher fellowships from CINSW and Tour de Cure.

## Conflict of Interest

The authors declare that the research was conducted in the absence of any commercial or financial relationships that could be construed as a potential conflict of interest.

## Publisher’s Note

All claims expressed in this article are solely those of the authors and do not necessarily represent those of their affiliated organizations, or those of the publisher, the editors and the reviewers. Any product that may be evaluated in this article, or claim that may be made by its manufacturer, is not guaranteed or endorsed by the publisher.
