# Supplementary material for: Systematic In Vitro Evaluation of a Library of Approved and Pharmacologically Active Compounds for the Identification of Novel Candidate Drugs for KMT2A-Rearranged Leukemia
Source: Front Oncol. 2022 Jan 20;11:779859. doi: 10.3389/fonc.2021.779859 (PMC8811472; doi:10.3389/fonc.2021.779859)
Supplement: Supplementary file 1 [file DataSheet_1.docx]

Supplementary Material

# Supplementary Figures

**Supplementary Figure 1.** **(A)** 2-Cl-ATP induces apoptosis in PER-485 cells. Representative plots (left) and quantification (right) of mean percentage increases of annexin V positive cells relative to vehicle-treated cells, based on flow cytometric determination of annexin V/7AAD staining in PER-485 cells. The results are expressed as the mean ± SE of three independent experiments. The significance of increases in annexin V positive cells was assessed by one sample t-tests. Asterisks represent significance levels of P-values. **, *p*<0.01. **(B)** Dose response curves for 2-Cl-ATP, cladribine and 2-CADO in KMT2A-r leukemia cell lines divided based on their sensitivity to 2-Cl-ATP.


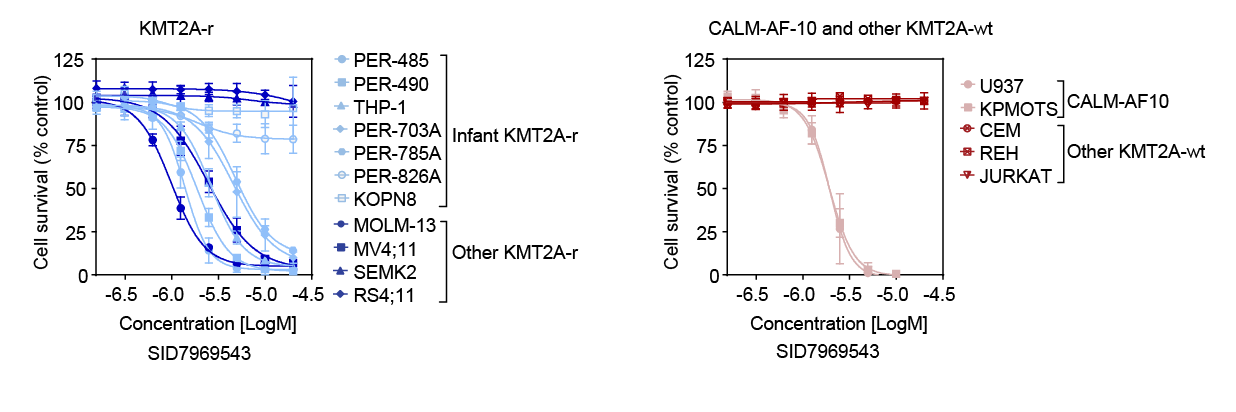


**Supplementary Figure 2.** Dose response curves for SID7969543 across a range of KMT2A-r, *CALM-AF10* translocated and KMT2A-wt leukemia cell lines as measured in 72-hour resazurin-based viability assays. The results are expressed as the mean % viability (relative to vehicle control-treated cells) ± SE of three independent experiments.


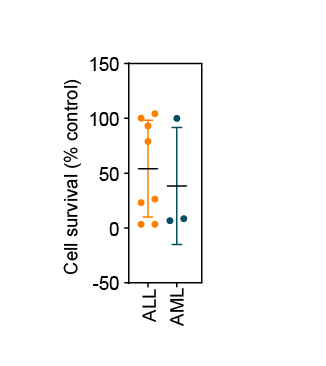


**Supplementary Figure 3.** Comparison of the viabilities of tested ALL versus AML cell lines (mean ± SE) as measured in 72-hour resazurin-based viability assays upon treatment with 10µM SID7969543.


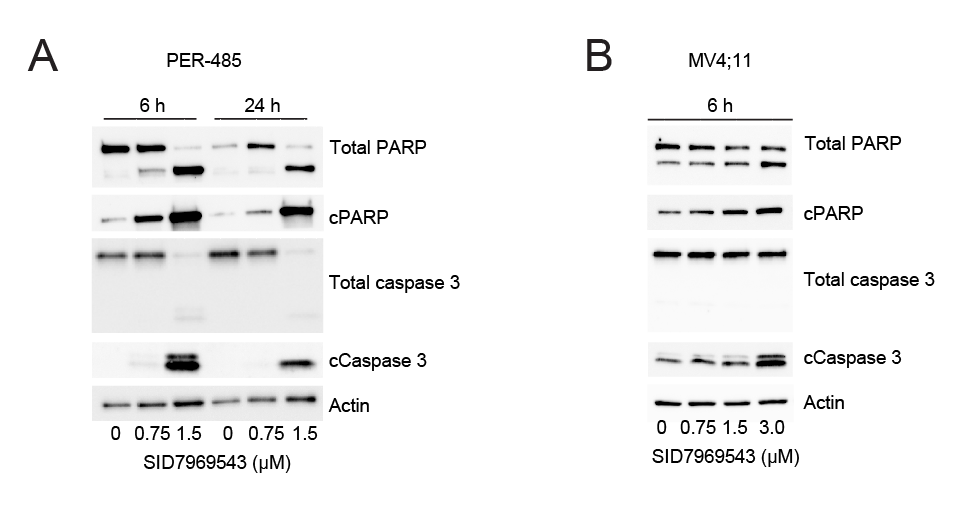


**Supplementary Figure 4.** Western blots showing induction of apoptotic markers cleaved PARP (cPARP) and cleaved caspase 3 (cCaspase 3) in KMT2A-r leukemia cell lines PER-485 and MV4;11 after treatment with SID7969543.

**Supplementary Figure 5.** Levels of *NR5A1* mRNA in KMT2A-r versus KMT2A-wt leukemia patients and cell lines. **(A)** Each data point represents the mRNA expression level of one infant patient based on the microarray dataset extracted from Stam *et al*. (2010). **(B)** Each data point represents the mRNA expression level of one cell line (relative to that of the PER-485 cells) as determined by qRT-PCR. Infant KMT2A-r cell lines: PER-485, PER-490, THP-1, PER-703 and KOPN8; KMT2A-wt cell lines: CEM, REH, Jurkat, Kasumi, KG1 and Loucy. The P-values were determined by unpaired t-tests. **(C)** Correlation analysis of the percentage of cell survival at 5 µM of SID7969543 (as measured in 72-hour resazurin-based viability assays) and *NR5A1* mRNA expression level in a panel of KMT2A-r and KMT2A-wt leukemia cell lines.


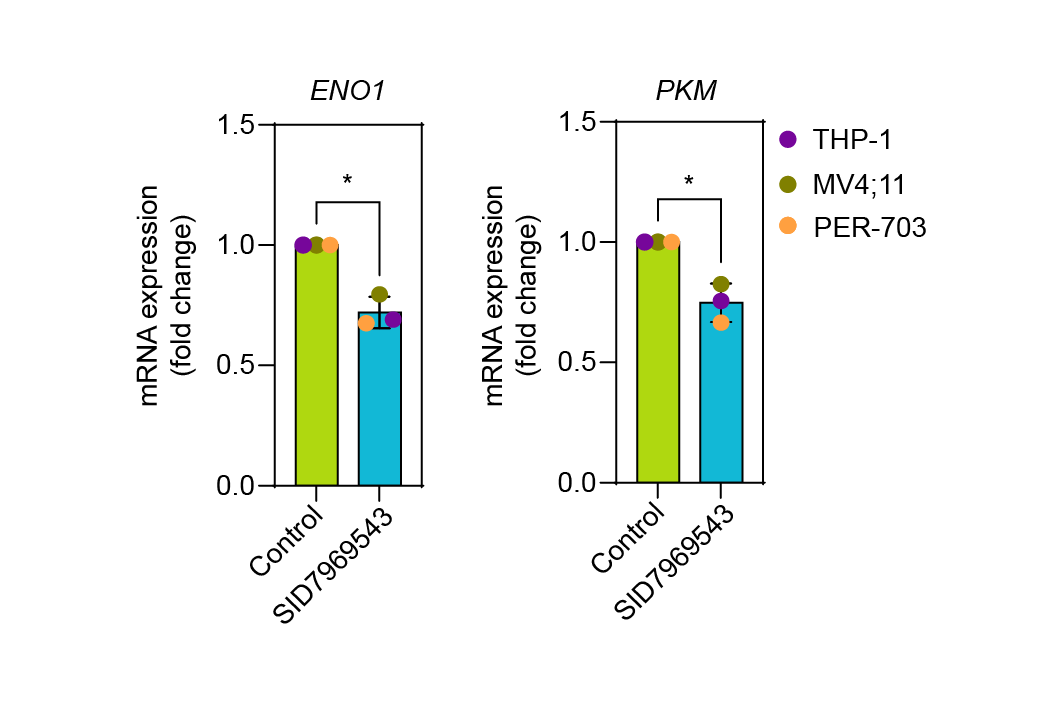
**Supplementary Figure 6.** mRNA expression level of NR5A1 target genes *ENO1* and *PKM* after three hours of SID7969543 treatment of three KMT2A-r leukemia cell lines, THP-1, MV4;11, and PER-703 (at 2.6 µM, 1.4 µM and 5 µM SID7969543, respectively). Statistical significance of mRNA expression changes was determined by one sample t-test. Asterisks represent significance levels of P-values. *, *p*<0.05.


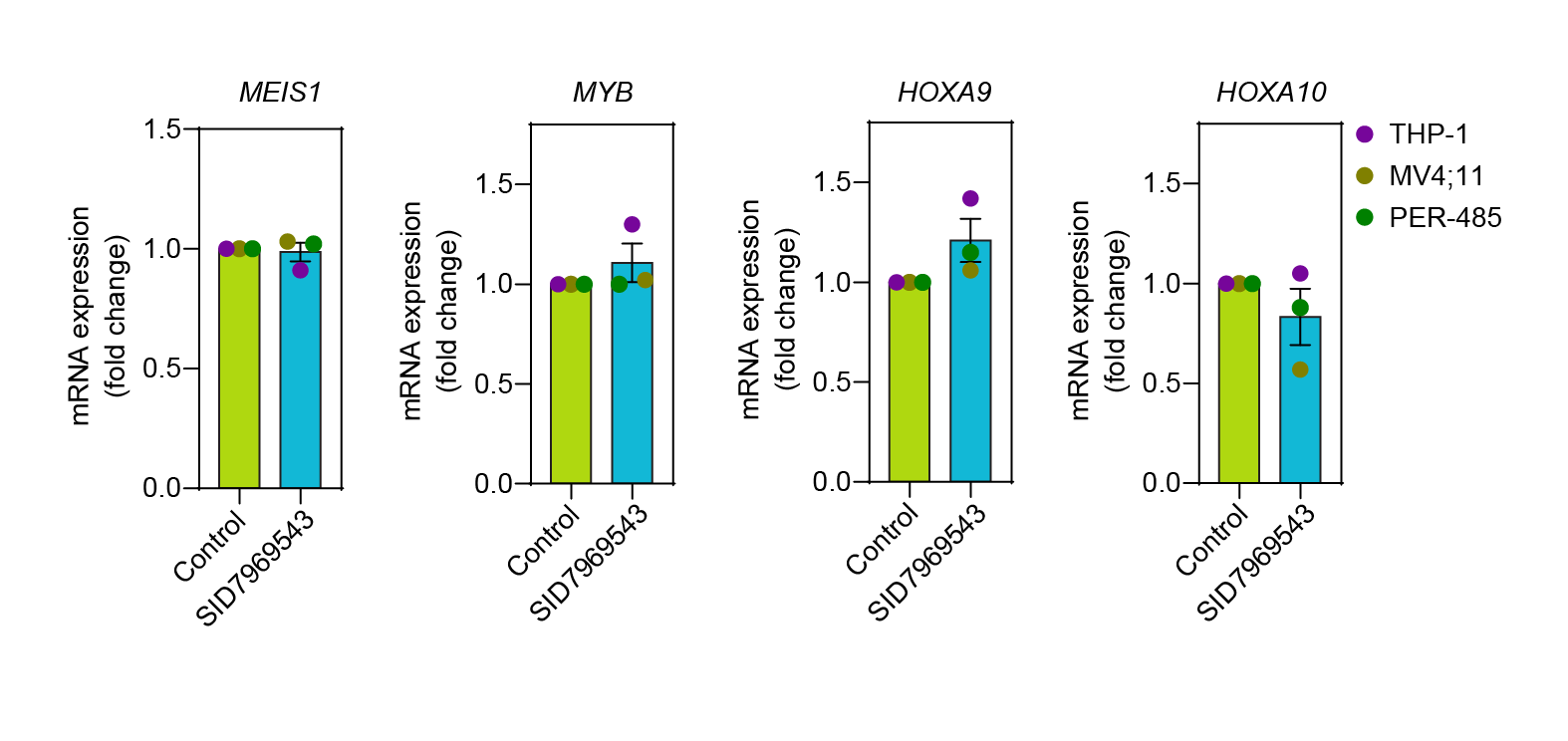


**Supplementary Figure 7.** mRNA expression level of KMT2A target genes *MEIS1*, *MYB*, *HOXA9*, and *HOXA10* in three KMT2A-r leukemia cell lines, THP-1, MV4;11, and PER-485 after 2.6 µM, 1.4 µM and 5 µM SID7969543 treatment, respectively, for three hours.


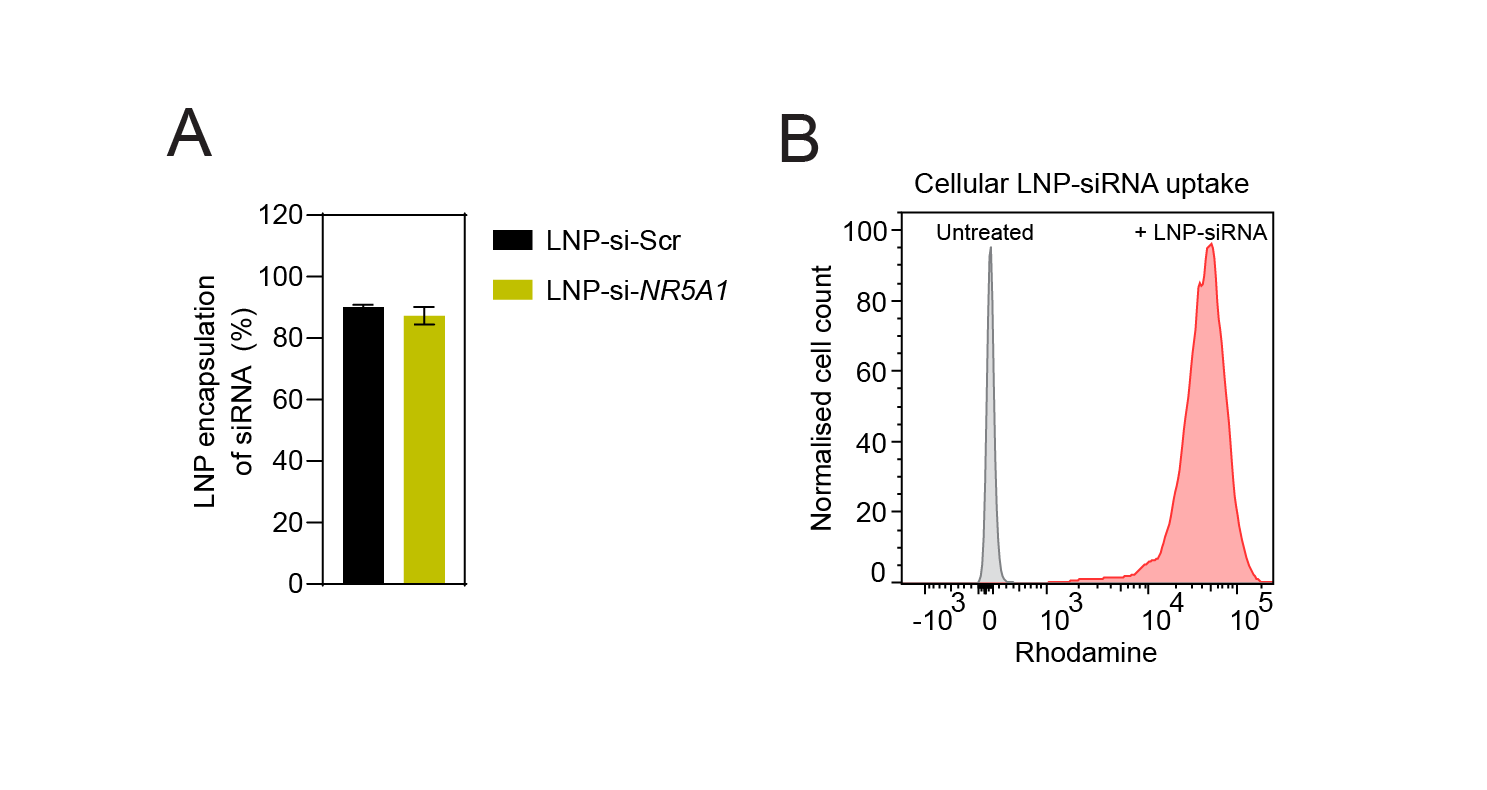
 **Supplementary Figure 8. (A)** Percentages of lipid-based nanoparticle (LNP) encapsulation of scrambled siRNA (si-Scr) or siRNA targeting *NR5A1* (si-*NR5A1*). Graphs represent mean ± SE of three independent experiments. **(B)** Flow cytometry histogram showing cellular uptake of fluorescently labelled LNP-siRNA after addition of LNP-siRNA to the cell suspension.


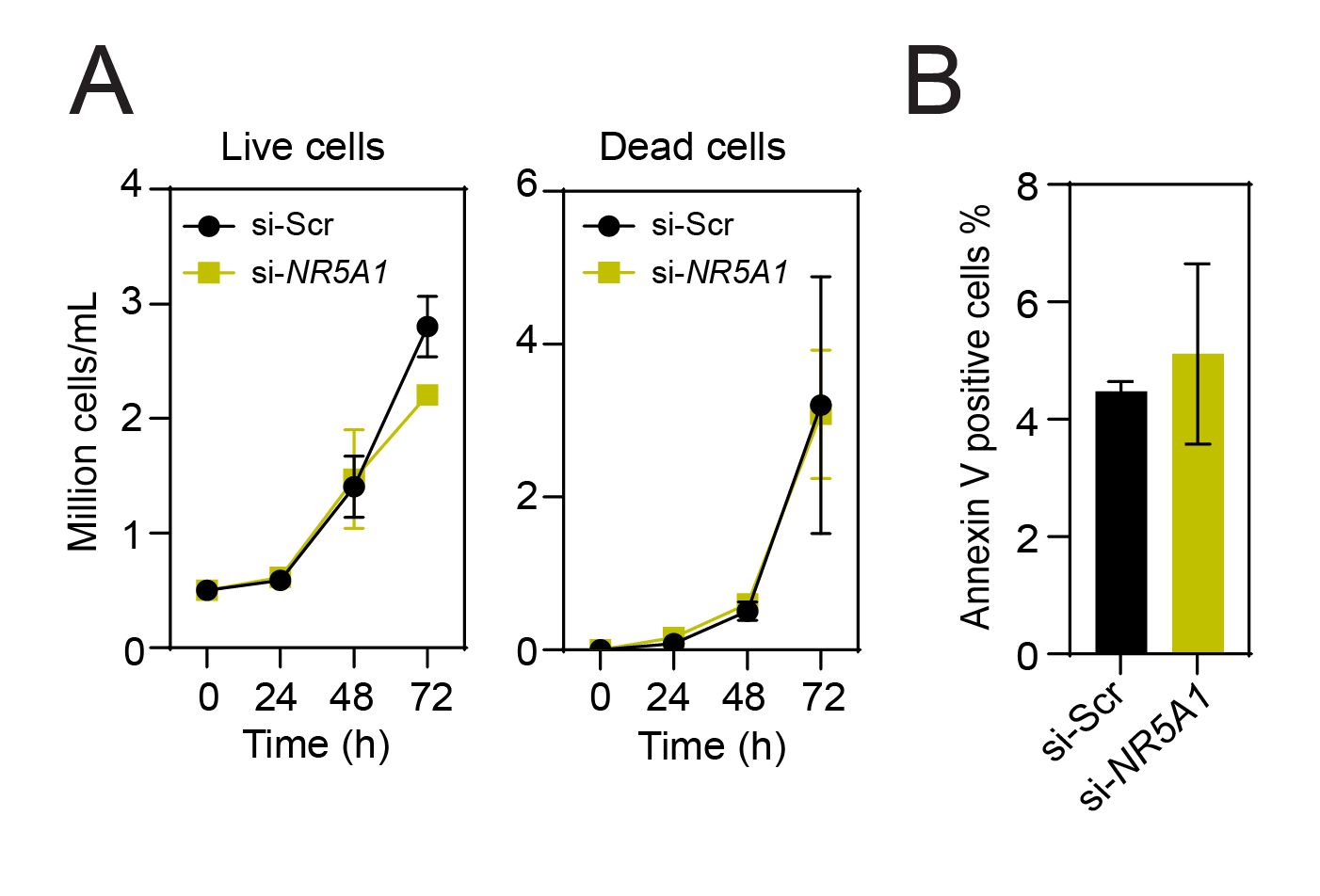
 **Supplementary Figure 9. (A)** Cell growth of KMT2A-wt REH cells as determined by trypan blue cell count after treatment with LNP-si-Scr (scrambled control) or LNP-si-*NR5A1* up to 72h. **(B)** Percentage of annexin V positive REH cells after a 48-hour exposure to LNP-si-Scr or LNP-si-*NR5A1*. Graphs represent mean ± SE of two independent experiments.


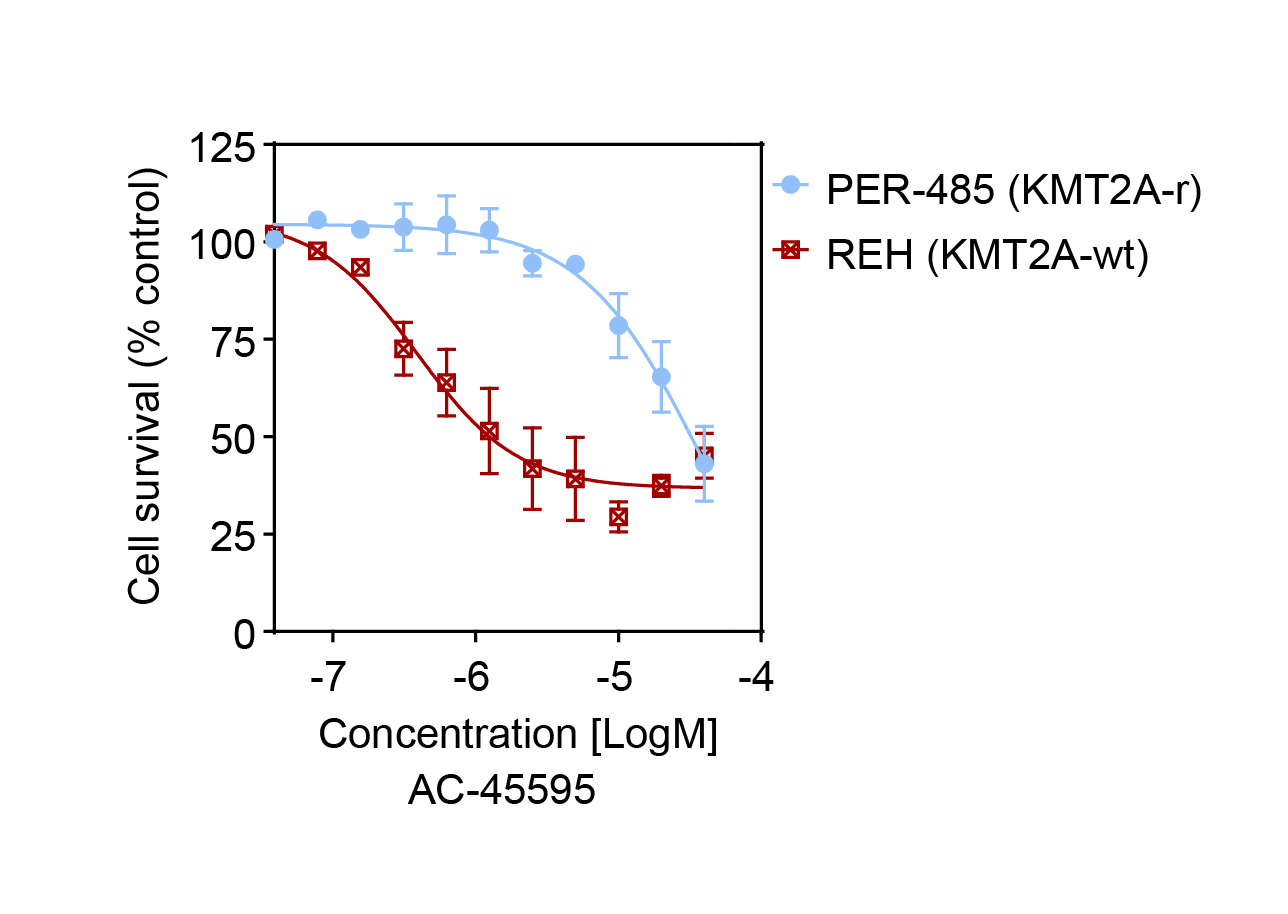


**Supplementary Figure 10.** Dose response curves for AC-45595 for the KMT2A-r leukemia cell line, PER-485, and KMT2A-wt REH cells as measured in a 72-hour resazurin-based viability assay. Data are expressed as the mean % viability (relative to vehicle control-treated cells) ± SE of three independent experiments.


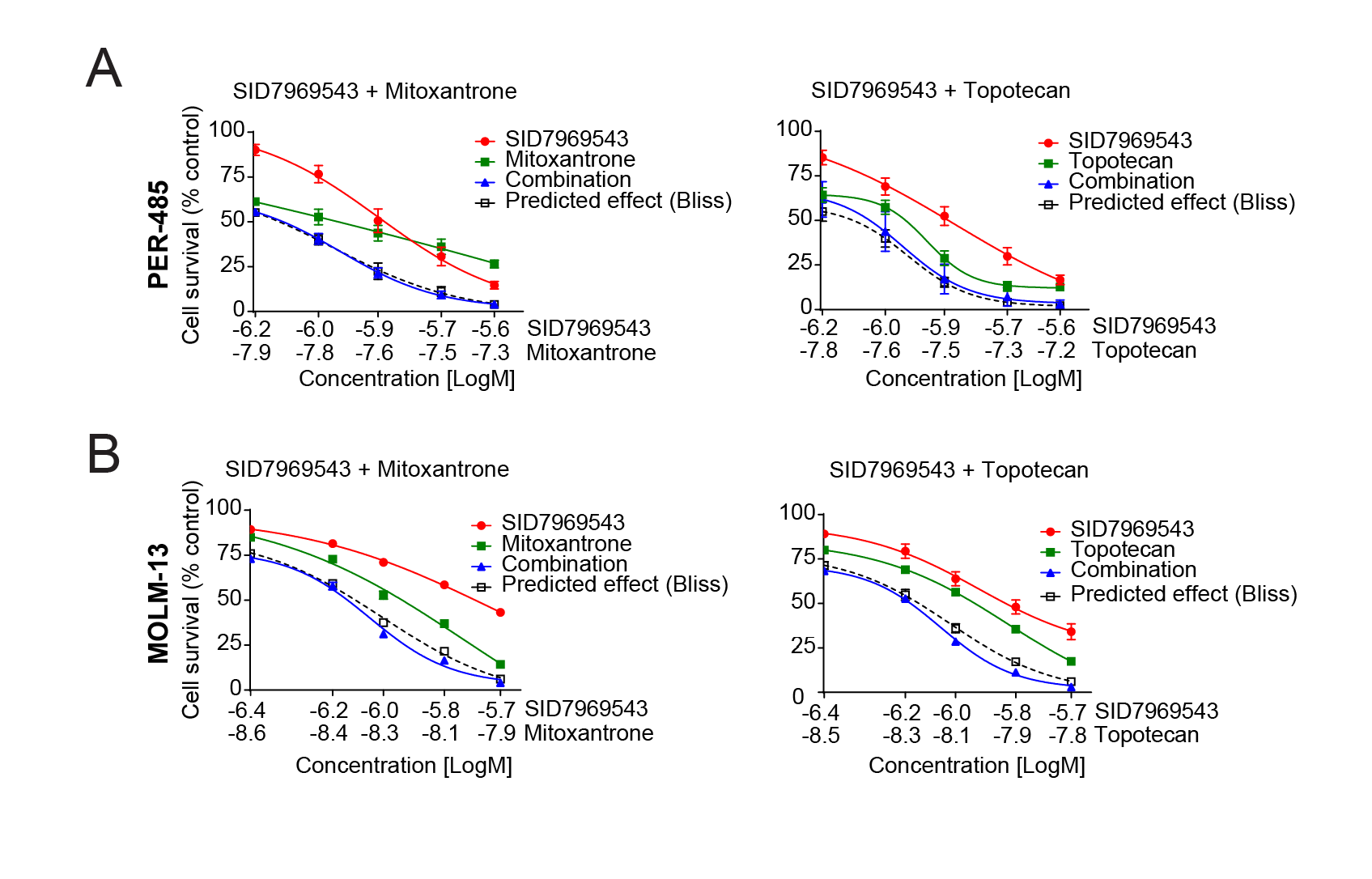


**Supplementary Figure 11.** Dose response curves for combination treatments of SID7969543 and standard-of-care chemotherapy drugs, mitoxantrone and topotecan in PER-485 **(A)** and MOLM-13 **(B)** determined by 72-hour resazurin-based viability assay. Drug synergy was calculated by applying the Bliss additivity model. Dotted lines indicate predicted viability when the drugs have additive effects.

**Supplementary Figure 12.** Prediction of metabolic stability of SID7969543 by mouse microsomal stability assays. Data are depicted as the mean ± SE of two independent experiments.

## Supplementary Tables

**Supplementary Table 1: Cell line information**

|  | **Cell line** | **Translocation** | **Disease** | **2-Cl-ATP  IC_50_ (µM)** | **SID7969543  IC_50_ (µM)** |
| --- | --- | --- | --- | --- | --- |
| **CALM-AF10** | U937 | CALM-AF10 | AML | 3.5 | 1.9 |
|  | KP-MO-TS | CALM-AF10 | AML | 4.4 | 1.9 |
| **KMT2A-r** | PER-485 | t(4;11) | derived from infant ALL; mixed lymphoid/myeloid phenotype | 2.5 | 1.4 |
|  | PER-490 | t(4;11) | Infant ALL | 4.5 | 1.9 |
|  | THP-1 | t(9;11) | Infant AML | >20 | 2.6 |
|  | PER-703 | t(1;11) | derived from infant ALL; mixed lymphoid/myeloid phenotype | 4.3 | 4.9 |
|  | PER-785 | t(4;11) | Infant ALL | 3.5 | 5.0 |
|  | PER-826 | Complex, t(11;19) | Infant ALL | 2.6 | >20 |
|  | KOPN-8 | t(11;19) | Infant pre-B cell ALL | 13.4 | >20 |
|  | MOLM-13 | t(9;11) | AML | 5.6 | 1.0 |
|  | MV4;11 | t(4;11) | Childhood AML | 2.7 | 2.5 |
|  | SEMK2 | t(4;11) | Pre-B cell childhood ALL | 5.1 | >20 |
|  | RS4;11 | t(4;11) | Pre-B cell ALL | 5.9 | >20 |
| **Other KMT2A-wt** | CEM | - | Childhood T-cell ALL | 11.4 | >20 |
|  | REH | - | Pre-B cell ALL | >20 | >20 |
|  | Jurkat | - | Childhood T-cell ALL | >20 | >20 |
| **Solid tumors** | KELLY | - | Neuroblastoma | >20 | >20 |
|  | BE(2)C | - | Childhood neuroblastoma | >20 | >20 |
|  | HEY | - | Ovarian carcinoma | >20 | >20 |
|  | 27/87 | - | Endometrioid ovarian cancer | >20 | >20 |
|  | MCF-7 | - | Breast adenocarcinoma | >20 | >20 |
|  | H460 | - | Lung carcinoma | >20 | >20 |
|  | LNCaP | - | Prostate carcinoma | 14.9 | >20 |
| **Non-malig-nant cells** | MRC-5 | - | Normal lung | >20 | >20 |
|  | WI-38 | - | Normal lung | >20 | >20 |
| IC_50_: inhibitory concentration resulting in 50% reduction of cell survival relative to control, IC_50_ values were derived from three independent experiments; ALL: acute lymphoblastic leukemia; AML: acute myeloid leukemia. | | | | | |

**Supplementary Table 2: Antibodies for Western blotting**

| **Name** | **Manufacturer** | **Catalogue number** | **Dilution** |
| --- | --- | --- | --- |
| **PARP (46D11)** | Cell Signaling Technologies | 9532 | 1:1000 |
| **Cleaved PARP (Asp214)** | Cell Signaling Technologies | 9541 | 1:1000 |
| **Caspase3 (D3R6Y)** | Cell Signaling Technologies | 14220 | 1:1000 |
| **Cleaved Caspase-3 (Asp175)** | Cell Signaling Technologies | 9664 | 1:1000 |
| **Actin** | Sigma-Aldrich | A2066 | 1:2000 |
